# Psychological risk factors of addiction to social networking sites among Chinese smartphone users

**DOI:** 10.1556/JBA.2.2013.006

**Published:** 2013-04-12

**Authors:** Anise M. S. Wu, Vivi I. Cheung, Lisbeth Ku, Eva P. W. Hung

**Affiliations:** ^1^University of Macau, Macau, China; ^2^Centennial College, Hong Kong, China

**Keywords:** social networking site, addiction, social cognitive theory, impulsivity, smartphone, Chinese

## Abstract

*Background and aims:* Smartphones allow users to access social networking sites (SNSs) whenever and wherever they want. Such easy availability and accessibility may increase their vulnerability to addiction. Based on the social cognitive theory (SCT), we examined the impacts of outcome expectancies, self-efficacy, and impulsivity on young Chinese smartphone users' addictive tendencies toward SNSs. *Methods:* Two hundred seventy-seven Macau young smartphone users (116 males and 161 females; mean age = 26.62) filled out an online Chinese questionnaire concerning their usage of social networking sites via smartphones, addiction tendencies toward SNSs, impulsivity trait, outcome expectancies toward the use, and Internet self-efficacy. *Results:* The findings revealed that those who spent more time on SNSs also reported higher addictive tendencies. Addictive tendencies were positively correlated with both outcome expectancies and impulsivity, but negatively associated with Internet self-efficacy. These three psychological variables explained 23% of the variance in addictive tendencies. *Conclusions:* The findings of this study suggest that, compared to demographics, psychological factors provide a better account for addictive tendencies towards SNSs among Chinese smartphone users in Macau. The three psychological risk factors were low Internet self-efficacy, favorable outcome expectancies, and high impulsivity trait. Educational campaigns with screening procedures for high-risk groups are recommended for effective prevention and treatment.

## Introduction

Access to the Internet is increasingly easy due to advances in mobile technology and the prevalence of smartphones. In Macau, a Special Administrative Region (SAR) of the People's Republic of China, the number of 3G smartphone users increased from about 8% of the population in 2008 to over 95% in 2012 (Bureau of Telecommunications Regulations of Macau SAR Government, 2012). This dramatic spread of smartphones is accompanied by the ever increasing popularity of social networking sites (SNSs), particularly among young adults (Subrahmanyama, Reich, Waechter & Espinoza, 2008). To these days, SNSs such as Facebook can be easily accessed by not only default Internet browsers on smartphones but also some free smartphone applications (“app”) of SNSs. However, a small but growing number of empirical studies have shown that SNS use is potentially addictive, and that this behavioral addiction can lead to clinically significant adverse consequences (see review by Kuss & Griffiths, 2011). It is likely that with the enhanced accessibility of SNSs via smartphones, susceptibility to such addiction may also be on the rise. Matthews, Pierce and Tang (2009) have provided some initial evidence to support that smartphone users have greater potential of developing addiction towards web-based social interaction. It is therefore the purpose of the present study to empirically examine the psychological risk factors of addiction to SNSs among Chinese smartphone users.

### Use of and addiction to SNSs

SNSs are rapidly becoming global consumer phenomenon. It was estimated that between 55% and 82% of teenagers and young adults use SNSs on a regular basis (Kuss & Griffiths, 2011). Through SNSs such as Facebook, young people aim to obtain information, gain entertainment, and maintain personal status (Park, Kee & Valenzuela, 2009; Pempek, Yermolayeva & Calvert, 2009). But most important of all, they use SNSs for social purposes such as communicating with their peers, gaining social compensation, and getting social identity gratifications (Kuss & Griffiths, 2011). Some of the commonest SNS activities among young people are reading and responding to posts on their walls (pages), and browsing their friends' walls (pages) (Subrahmanyama et al., 2008).

While SNSs allow for relationship building in a brand new format, their usage can be excessive, or even compulsive in some cases. For example, Subrahmanyama et al. (2008) found that some college respondents spent up to four or more hours on SNSs everyday. Problematic use of SNSs is also related to a host of negative psychosocial outcomes such as decreased real-life community involvement, poorer academic performance, and more relationship problems (Blaszczynski, 2006; Kuss & Griffiths, 2011).

As in other types of Internet addiction, some Internet users develop a specific addiction to SNSs, rather than to the medium (i.e., the Internet) *per se* (Kuss & Griffiths, 2011). Individuals with addictive tendencies toward SNSs may show addiction symptoms such as loss of control and experiencing withdrawal (Wilson, Fornasier & White, 2010). Based on Young's criteria of Internet addiction (1996), other symptoms of addictive tendencies toward SNSs may also include tolerance development, being preoccupied with SNSs, deception to others about the usage, mood-regulatory purpose, and adverse consequences such as loss of significant relationships, jobs, and educational or career opportunities (Kuss & Griffiths, 2011).

Compared to other forms of Internet addiction such as cybersexual addiction, addiction to SNSs does not command sufficient research concerns. Little is known about the risks of the addiction, nor about the protective factors that could guard against this specific form of addiction. Such knowledge, however, is essential for better understanding of the addiction, how it is similar to or distinctive from other types of Internet addiction. Such knowledge will also inform more effective intervention of problematic use of SNSs, and prevention of the development of addiction to SNSs.

### Conceptual framework of the present study

Based on the social cognitive theory (SCT; Bandura, 1986, 1989), the present study examines whether two cognitive factors – outcome expectancies toward SNSs and Internet self-efficacy – and one personality factor – impulsivity – are associated with the heightened risk of developing addictive tendencies toward SNSs among young Chinese smartphone users in Macau.

*The social cognitive theory*. The SCT posits that human behavior can be explained by the triadic and reciprocal causation of personal factors, behavior, and environment. The two major personal determinants of a behavior are perceived outcome expectancies and self-efficacy toward that behavior. Outcome expectancy is generally defined as expectation of behavioral consequences of a specific personal action, whereas self-efficacy is the individual's belief about his/her own ability to perform and execute that action.

According to the SCT, people are more likely to engage in behaviors which they anticipate to have positive outcomes or rewards than those they perceive otherwise (Bandura, 1982; Compeau & Higgins, 1995). Positive outcome expectancies have been found to be associated with higher frequency of addictive behaviors among young people such as drinking and gambling (e.g., Shell, Newman & Xiaoyi, 2010; Wickwire, Whelan & Meyers, 2010). In the same vein, Internet use is generally reinforced by positive outcomes of the use, such as feelings of well-being in relation to others (Chakraborty, Basu & Kumar, 2010). Tsai and Lin (2001) also found a positive correlation between perceived usefulness of the Internet and Internet addiction symptoms among Taiwanese adolescents. Regarding the SNS use, people generally perceive/expect several functions of using SNSs, including communication, socialization, entertainment, information, and identity development/maintenance (Dunne, Lawlor & Rowley, 2010; Park et al., 2009). It is hypothesized that those who expect more positive outcomes from using SNSs tend to spend more time on the sites via smartphones and to report higher addictive tendencies (Hypothesis 1).

Self-efficacy is another prominent predictor of actual behaviors in the theoretical framework of the SCT because an individual will carry out a specific behavior only if they evaluate themselves to be competent in enacting it. In the domain of Internet use, basic skills such as establishing stable Internet connection and manipulating information on the Internet are needed for the successful use of various Internet applications including SNSs (Eastin & LaRose, 2000). Internet self-efficacy is therefore defined as a set of personal beliefs regarding one's ability to successfully use Internet tools to achieve different purposes such as searching for information and conducting instant communication (Eastin & LaRose, 2000).

Consistent with the SCT, previous findings showed a positive correlation between Internet self-efficacy and Internet use. For instance, both American and Chinese young adults with higher Internet self-efficacy use the Internet more frequently (Eastin & LaRose, 2000; Shi, Chen & Tian, 2011). Hong Kong teenagers who perceived themselves as more information literate also reported more Internet addiction symptoms (Leung & Lee, 2012). Among the small handful of studies on SNSs, Baker and White (2010) found that Australian adolescents' perceived behavioral control over regular SNS visits predicted how frequently they visited SNSs in one week. Nevertheless, Pelling and White (2009) observed that young people's specific perceived efficacy of SNS use did not significantly predict their actual high usage of SNSs (i.e., over four unique visits to SNSs per day). They argued that the specific efficacy construct of the use of SNSs might only reflect accessibility to SNSs but not their perceived competence in actually using SNSs. Therefore, in the present study, we examined the generalized self-efficacy of Internet use, instead of specific self-efficacy of SNS use. Individuals with low Internet self-efficacy may perceive various Internet tools as difficult to use and thus are more likely avoid using Internet applications such as SNSs. It is therefore hypothesized that people with higher Internet self-efficacy use SNSs more frequently and report higher addictive tendencies (Hypothesis 2).

*Impulsivity.* The SCT assumes humans as rational beings who take action after cognitively evaluating the potential gains of an action and their own competence of the action. But human behaviors are often influenced by less conscious and rational factors such as affective states and personality traits. Regarding the use of SNS, previous studies have found several personality correlates such as low self-esteem/identity, narcissism, and neuroticism (Kuss & Griffiths, 2011; Pelling & White, 2009). However, these traits typically have low explanatory power for addictive tendencies toward SNSs. For instance, Wilson et al. (2010) showed that the big five personality traits (i.e., openness, conscientiousness, extraversion, agreeableness, and neuroticism) and self-esteem explained less than 10% of the variance in SNS use and its additive tendencies among Australian young adults. This is why in the present study we chose not to study general personality traits, but impulsivity – a specific personality trait that had been identified as a salient dispositional risk factor of numerous addictive behaviors such as substance abuse, pathological gambling, and Internet addiction (e.g., Beard & Wolf, 2001; Cao, Su, Liu & Gao, 2007; Spinella, 2007; Tang & Wu, 2012).

Impulsivity is characterized by behavioral disinhibition and need satisfaction without forethought (Kreek, Nielsen, Butelman & LaForge, 2005; Patton, Stanford & Barratt, 1995). Impulsive individuals possess a higher tendency to overeat, overspend, gamble, abuse drugs, engage in risky sex, and do or say things they regret (Cyders & Smith, 2008). Through smartphones, impulsive individuals can gain immediate access to SNSs, which may in turn fuel their addictive tendencies towards SNSs. Hence, it is hypothesized that impulsive people tend to visit SNSs more frequently via smartphones and report higher addictive tendencies (Hypothesis 3).

### The present study

Given the extreme wide use of smartphones in Macau, the immense popularity of SNSs among young adults, and the potential detrimental consequences of addiction to SNSs, it is essential to assess psychological risk factors of developing such an addiction among young smartphone users in Macau. To our best knowledge, none of the empirical research on Chinese people's use of and addictive tendencies to SNSs has been done. Based on the SCT, this study examined the potential impacts of two cognitive risk factors (i.e., outcome expectancies and Internet self-efficacy) on addiction to SNSs among young Chinese smartphone users. The role of impulsivity trait, a common dispositional characteristic among at-risk groups of various forms of behavioral addiction, was also investigated. The relative importance of these three psychological factors on addictive tendencies toward SNSs was also compared. The findings can provide insights on effective intervention programs for preventing and treating such addiction.

## Methods

### Participants

Young Chinese adults aged from 18 to 40 years are the target population of this study. In this study, 316 Chinese participants filled out an online questionnaire. However, 15 of them were excluded as they were not smartphone users. Another 24 participants were also excluded because of the age criterion. The final sample was therefore composed of 277 young adults of 116 males and 161 females (*M*_age_ = 26.62, *SD* = 4.42). The majority of them were single (77.1%), educated (71.8% having bachelor's degree or higher) and under employment (77.5%). Demographic information of the participants is listed in Table 1.

### Study procedure

Since addiction is a sensitive topic with negative connotations, we collected data by an online survey application, SurveyMonkey (www.surveymonkey.com), in order to minimize possible social desirability effect. The survey hyperlink was posted on Facebook as an event for one month because Facebook is the most popular SNS in Macau. Potential participants were recruited by convenience sampling, and they received invitation e-mails or messages from the researchers by snow balling techniques. They could click on the hyperlink and were redirected to the webpage of the online Chinese questionnaire. Before filling out the questionnaire, participants were instructed to carefully read the objectives of the study and their rights regarding their voluntary participation. Anonymity and confidentiality were assured. Informed consent was then obtained. A pilot test on the comprehensibility of the self-administered questionnaire and the fluency of response entry had been conducted by three Chinese young adults in Macau before the actual implementation of the survey. This study adheres to the guidelines for research ethics of the University of Macau and meets all the ethical requirements.

**Table 1.** Demographic characteristics of the participants (*N* = 277)

		*N*	%
Age	20 years or below	16	5.7
	21-25 years	110	39.8
	26-30 years	109	39.4
	31-35 years	30	10.8
	36-40 years	12	4.3
Gender	Male	116	41.9
	Female	161	58.1
Education level	Primary	2	0.7
	Junior secondary	8	2.9
	Senior secondary	68	24.6
	Two-year certificate	8	2.9
	Bachelor	160	57.8
	Master or higher	39	14.0
Work status	Student	61	22.5
	Employed	210	77.5
Marital status	Single	212	77.1
	Married	63	22.9
Usage of SNSs via smartphones (per day in past 3 months)	None	3	1.1
	1 hour or less	107	38.9
	1-2 hours	101	36.7
	3-4 hours	33	12.0
	5-6 hours	10	3.7
	7 hours or above	21	7.6

### Measures

The online questionnaire included two major parts. The first part was a demographic section. Participants were asked to fill out their information on gender (0 = male; 1 = female), age, marital status (1 = single; 2 = married), work status (1 = student; 2 = employed), educational level (0 = lower than bachelor; 1 = bachelor or higher), and the average number of hours spent on SNSs via smartphones per day.

In the second part, participants were asked to honestly record their answers to the items of four measurement inventories. For all four inventories, higher scores represent higher endorsement of the psychological features assessed. First, similar to previous research on online game addiction (Qin, Rao & Zhong, 2007), addictive tendencies toward SNSs were assessed by a 20-item inventory which was modified from Young's Internet Addiction Test (IAT, 1998). A similarly modified Chinese version of the scale has been used to assess video game addiction among Chinese young adults with satisfactory reliability (Wu, Lei & Ku, in press). Respondents were asked to report how often they experienced obsession, compulsion, or problems related to the use of SNSs, on a 5-point Likert scale (1 = never to 5 = always). Possible total scores range from 20 to 100. Based on Wang et al.'s study on Chinese students' Internet use (2011), we adopted similar cut-off points of normal users (scores 20–49) and probable problematic users (scores 50 or above). A sample item was “How often do you try to cut down the amount of time you spend on SNSs and fail?” In this study, the reliability of this scale was .92.

Positive outcome expectancies of SNS use were measured by the modified 19-item *Outcome Expectancy of Internet Use Questionnaire* (Cheong, 2001). Respondents were asked to state their agreement on each potential outcome of using SNSs, on a 5-point Likert type scale (1 = strongly disagree to 5 = strongly agree). Thirteen items described positive expectancies (e.g., “People who use SNSs have more opportunities to communicate with others”), whereas six items stated negative expectancies (e.g., “People who use SNSs put their privacy at risk”). The negative items were recoded before the computation of the mean score of the scale for each respondent. One of them (“SNSs are addictive”) was deleted from the scale because it had negative item-total correlation, and the Cronbach's alpha for the resultant scale was .72.

Internet self-efficacy was measured by seven items of the *Internet Self-Efficacy Scale* (Eastin & LaRose, 2000), with a 5-point Likert scale (1 = strongly disagree to 5 = strongly agree). Sample item was “I feel confident describing functions of the Internet hardware”. Impulsivity was measured by the Chinese version of 19-item Eysenck and Eysenck's impulsiveness scale (1991), which has been used to assess Chinese college students' impulsivity (Tang, Chua & Wu, 2011) with a yes/no response format. The scale score was computed by summing all affirmative responses. A sample item of this scale was “Do you often do things on the spur of the moment?” The Cronbach's alpha for the scales of Internet self-efficacy and impulsivity were .60 and .78, respectively, in this study.

## Results

SPSS Statistics 19.0 was used for the statistical analyses, including descriptive statistics, correlation analysis, and regression analysis. Only 1% of the participants (*n* = 3) had not visited SNSs via smartphones in the previous three months at the time of the survey. The majority of the participants estimated their usage to be two hours or less: about two-fifth (*n* = 107) reportedly spent less than one hour per day, while 37% (*n* = 101) spent one to two hours. There were 23% of the participants spending three hours or more (*n* = 64). The usage of SNSs was not significantly associated with any other demographics (all *p*s > .05). About 12% (*n* = 33) scored 50 or above on the addictive tendencies scale and were classified as probable problematic users. These users were more likely to be students (c^2^(1) = 6.14, *p* < .05), and had lower educational attainment than normal users (c^2^(1) =3.77, *p* = .05). As expected, those with higher addictive tendencies also visited SNSs via smartphones more frequently (*r* = .31, *p* < .01). The results of correlation analyses were presented in Table 2.

Correlation analyses were used in hypothesis testing, and the results supported Hypotheses 1 and 3. First, we observed that outcome expectancies was positively correlated with both addictive tendencies and daily usage (*r*s = .21 and.18, respectively, *p*s < .01), as did impulsivity (*r*s = .23 and.14, respectively, *p*s < .05). However, there was a negative correlation between Internet self-efficacy and addictive tendencies (*r* = –.42, *p* < .01), whereas the correlation between Internet self-efficacy and daily usage was statistically non-significant (*p* > .05). Consistently, probable problematic users tended to report more positive outcome expectancies, higher impulsivity, but lower Internet self-efficacy (*r*s ranged from .14 to .23, *p*s < .05). These findings provided support to Hypotheses 1 and 3 but were contrary to Hypothesis 2.

Hierarchical regression analyses were conducted to test the relative explanatory values of three sets of variables on addictive tendencies: (1) the demographics (gender, age, marital status, education level, and work status); (2) the personality factor (i.e., impulsivity trait); and (3) the two cognitive factors of the SCT (i.e., outcome expectancies and Internet-self-efficacy). The demographic factors were first controlled in Block 1. Impulsivity was entered in Block 2, and the two cognitive factors were entered in Block 3.

Block 1 brought only marginally significant explanatory value to addictive tendencies (*F*(5, 254) = 2.16, *p* = .059), and none of the demographic variables emerged as a significant factor in Block 1. After controlling for all demographics, both Blocks 2 and 3 significantly explained the variance in the total score of addictive tendencies (*F*(1, 253) = 15.69 and *F*(2, 251) = 29.19, *p*s < .001). The overall *R*^2^ of the regression model was .27 (*F*(8, 251) = 11.44, *p* < .001). The final model of the regression analysis (see Table 3) revealed that Internet self-efficacy (beta = –.39, *p* < .01), outcome expectancies (beta = .17, *p* < .01), impulsivity (beta = .11, *p* =.07), and work status (beta = –.14, *p* < .05) significantly explained the variance in addictive tendencies. The results suggest that low Internet self-efficacy, positive outcome expectancies, and high impulsivity constituted the potential risk factors of addictive tendencies to SNSs.

Since preliminary analyses showed some significant associations between demographic variables and addictive tendencies, the potential moderating effects of the demographics on the relationship between the three psychological factors and addictive tendencies were further explored. These moderating effects were tested by five different hierarchical regression analyses. Each regression model involved the same three blocks mentioned above and an additional block of interaction terms formed by one demographic variable and the three psychological factors. In sum, no significant moderating effects of gender, age, marital status, education, and work status were observed (*F* Change(3, 248) = 1.16, .88, 1.54, 1.01, and 1.42 respectively, *p*s > .05).

**Table 2.** Means, standard deviations, and correlations among variables (*N* = 277)

	Variables	Mean	*SD*	1	2	3	4	5	6	7	8	9	10
1.	Addictive tendencies	37.09	11.23	−									
2.	Probable problematic user or not#	−	−	.73^**^	−								
3.	Daily usage of SNSs via smartphones	3.01	1.18	.31^**^	.23^**^	^–^							
4.	Internet self-efficacy	1.64	0.23	−.42^**^	−.23^**^	−.09	−						
5.	Outcome expectancies	3.37	0.50	.21^**^	.17^**^	.18^**^	−.10	−					
6.	Impulsivity	2.81	0.47	.23^**^	.14^*^	.14^*^	−.25^**^	.14^**^	−				
7.	Gender#	−	−	.03	[.10]	−.04	−.06	.11	−.28^**^	−			
8.	Age	26.62	4.42	−.20^**^	−.05	.02	.12	.01	−.05	−.18^**^	−		
9.	Marital status#	−	−	−.13^*^	[.06]	.05	.09	−.01	−.11	[2.71]	.54^**^	−	
10.	Educational level#	−	−	−.11	[3.77]^^^	−.08	.05	−.06	−.09	[.13]	.18^**^	[.46]	−
11.	Work status#	−	−	−.16^**^	[6.14]^*^	.03	−.03	.03	.00	[2.57]	.49^**^	[16.48]^**^	[14.16]^**^

*Note:* ^*^ *p* < .05; ^**^ *p* < .01; ^ *p* =.05; # Dichotomous variable; Chi-square value is reported inside square brackets.

**Table 3.** Results of the regression analysis with addictive tendencies as criterion variable (*N* = 277)

	Variables	Standardized coefficients (Beta)	*t*	*R^2^* change	*F* change
Model 1				.04	2.16
Block 1	Gender	−.01	−0.22		
	Age	−.13	−1.57		
	Marital status	−.02	−0.27		
	Educational level	−.05	−0.76		
	Work status	−.07	−1.03		
Model 2				.06	15.69
Block 1	Gender	.06	0.91		
	Age	−.13	−1.59		
	Marital status	.02	0.24		
	Educational level	−.02	−0.37		
	Work status	−.08	−1.16		
Block 2	Impulsivity	.25	3.96^**^		
Model 3				.17	29.19
Block 1	Gender	−.02	−0.34		
	Age	−.07	−1.00		
	Marital status	.01	0.21		
	Educational level	−.00	−0.07		
	Work status	−.14	−2.18^*^		
Block 2	Impulsivity	.11	1.85#		
Block 3	Internet self-efficacy	−.39	−6.89^**^		
	Outcome expectancies	.17	3.00^**^		

*Note:* ^*^ *p* < .05; ^**^ *p* < .01; # *p* = .07. The overall *R*^2^ of Models 1, 2, and 3 are .04, .10 and .27, respectively.

## Discussion

In the present study, almost all Chinese young adults surveyed had a smartphone and access to SNSs via it. Among our young smartphone users, nearly one-fourth of them spent three hours or more on SNSs everyday. Consistent with previous findings of Internet addiction (Shek, Tang & Lo, 2008; Wang et al., 2011), the present study demonstrated that daily usage of SNSs via smartphones was positively associated with addictive tendencies to SNSs. The findings also suggested that Chinese young adults, particularly students, are susceptible to addiction to SNSs.

Three psychological risk factors of addiction to SNSs were identified in this study. Among them, Internet self-efficacy was found to be the most salient factor of addictive tendencies among Chinese young adults. However, contrary to our hypothesis, it was negatively correlated to addictive tendencies. This finding suggests that people who are not self-efficacious of Internet use are more vulnerable to addiction to SNSs. This was inconsistent with previous findings by Leung and Lee (2012), who showed that the higher the perceived information technological skills (e.g., awareness of the latest information technology and products, abilities of locating information from multiple sources, and experiences in publishing ideas electronically), the more the Internet addiction symptoms. One plausible reason for these contradictory findings is that those who are information technologically skillful and competent with Internet tools are more likely to get “hooked” by other Internet activities than SNSs, which do not demand any advanced information knowledge and skills. Smartphones provide built-in and stable Internet connection, and smartphone applications (“apps”) of SNSs are designed to be very user friendly. Thus, accessing SNSs and using their socialization and entertainment functions via smartphones require very limited skills. These features may be particularly attractive to individuals who see themselves as lacking in Internet knowledge and skills for advanced Internet use. Therefore, young people who are only beginning to use the Internet, who possess low level of Internet related skills, and who perceive themselves as Internet non-competent, may be potentially vulnerable to problematic use of SNSs. The influence of an individual's Internet use history and preferred Internet activities can be further examined in future studies to test the above tentative explanation and whether it is a temporary phenomenon to Internet beginners. The present finding suggests that mental health professionals should pay attention to people who regard themselves as digitally incompetent, who potentially suffer a higher susceptibility to addiction to SNSs, which are both user friendly and psychologically rewarding.

Another cognitive risk factor from the SCT, outcome expectancies, was also found to significantly explain addictive tendencies. As hypothesized, young Chinese adults with more favorable outcome expectancies toward SNSs reported more time spent on SNSs via smartphones and higher addictive tendencies. Through SNSs, users usually expect to acquire the satisfaction of interpersonal relatedness via sharing their immediate thoughts and feelings. Like other kinds of Internet addiction, their visits to SNSs would be reinforced by positive outcomes through operant conditioning, and gradually an addiction towards these sites might be developed (Chakraborty et al., 2010). In this connection, the present finding suggests that mental health professionals may alter the unrealistically high expectancies of at-risk or problematic users to ease their addictive tendencies toward SNSs through cognitive behavioral therapy. For example, Whang, Lee and Chang (2003) observed that Internet addicts tended to create new social relationships online, and reported closer feelings for these online strangers than for their family and friends. However, they eventually felt lonelier because their need for belonging could not be satisfied by fleeting online relationships. Mental health professionals may discuss these findings with problematic users of SNSs to lower their expectations toward the positive impacts of SNSs on interpersonal need satisfaction, and provide behavioral training on reducing interpersonal difficulties that these users may face in real life. In addition, prevention campaigns of addiction to SNSs can also be used to educate young people of both short-term and long-term negative consequences of SNS use, such as addictive features of SNSs, privacy risk, and information overload. In particular, young people should be constantly reminded, through public advertisement and school education, that these negative consequences may outweigh the positive ones if their use of SNSs is excessive.

Consistent to the previous studies on other types of behavioral addiction (e.g., Spinella, 2007; Tang & Wu, 2012), impulsivity was shown to be another psychological risk factor of addictive tendencies to SNSs among Chinese smart-phone users in the present study. Impulsive individuals reported not only higher addictive tendencies but also more time spent on SNSs via smartphones. People with high impulsivity tend to initiate an action to satisfy their immediate needs without forethought (Patton et al., 1995). They are, therefore, particularly vulnerable to get addicted to SNSs, which may provide instant satisfaction of informational and relational needs via smartphones. Furthermore, people with high impulsivity often have problems with attention and are easily distracted by external stimuli (e.g., a pop-up signal of a new message in their profiles), and thus may be “tempted” to use SNSs even when they are studying or working. Since impulsive people tend to have lower self-awareness, reminders that serve to raise their awareness of excessive use may be useful. For example, a pop-up warning after every 30 minutes may help to alert them to the amount of time they have spent on the SNS. They may also be educated to use the built-in timer function of smartphones for monitoring their usage of SNSs and other apps. Proper screening procedures may also be conducted on school campus for identifying students with high impulsivity and vulnerability to addictions to SNSs, and educational seminars and workshops should be made available to them.

Three major limitations of the present study should be acknowledged. As with any self-report questionnaire survey, the findings of this study are susceptible to report bias and errors (e.g., inaccurate estimation of SNS use). In particular, the addictive tendencies toward SNSs were measured by a modified self-report assessment scale of Internet addiction, which should be regarded as only an external scale for reflecting the level of addictive tendencies rather than a clinical screening or diagnosis tool for addiction to SNSs. Moreover, the correlational design cannot test the causation effects of the psychological variables. The generalizability of the findings may also be limited, not only because participants were recruited through one single SNS, but also because all participants were young and well-educated adults residing in Macau. Longitudinal studies and multi-samples of different age and educational backgrounds are needed, so does the recruitment of more diverse users of other SNSs such as Twitter and Weibo. In addition, future studies of both smartphone and non-smartphone users are necessary in order to check for potential differences in the extent of risk and the influences of the risk factors among the two groups.

In conclusion, the present study expanded the literature to include smartphone use and addiction to SNSs. We identified three psychological risk factors of such addiction among young Chinese smartphone users. These three risk factors consisted of two cognitive and one personality attribute, which may heighten one's vulnerability to problematic use of SNSs. Based on the findings, educational campaigns with screening procedure of high-risk groups are recommended for intervention. The effectiveness of these campaigns should be further evaluated in future research.
